# CD4 T cells transduced with CD80 and 4-1BBL mRNA induce long-term CD8 T cell responses resulting in potent antitumor effects

**DOI:** 10.1186/2051-1426-2-S3-P264

**Published:** 2014-11-06

**Authors:** Hyun-Il Cho, Hye-Mi Park, Hyun-Jung Shon, Tai-Gyu Kim

**Affiliations:** 1 Catholic Hematopoietic Stem Cell Bank, Cancer Research Institute, College of Medicine, The Catholic University of Korea, Korea, Republic Of; 2 Dept. of Microbiology, Catholic Hematopoietic Stem Cell Bank, College of Medicine, The Catholic University of Korea, Korea, Republic Of

## 

Therapeutic cancer vaccines are an attractive alternative to conventional therapies to treat malignant tumors, and more importantly, to prevent recurrence after primary therapy. However, the availability of professional antigen-presenting cells (APCs) has been restricted by difficulties encountered in obtaining sufficient professional APCs for clinical use. We have prepared an alternative cellular vaccine with CD4 T cells that can be expanded easily to yield a pure and homogeneous population *in vitro*. To enhance their potency as a therapeutic vaccine, *in vitro *expanded CD4 T cells were transfected with RNAs encoding the costimulatory ligands CD80, 4-1BBL, or both (CD80-T, 4-1BBL-T, and CD80/4-1BBL-T cells, respectively). We observed augmented cell vitality in CD80/4-1BBL-T cells *in vitro *and *in vivo*. Significant CD8 T cell responses eliciting *in vivo *proliferation and cytotoxicity were obtained with CD80/4-1BBL-T cell vaccination compared to CD80-T and 4-1BBL-T cell vaccinations. Furthermore, CD80/4-1BBL-T cell immunization resulted in curing established EG7 tumors, resulting in the generation of memory CD8 T cell responses, and elicited therapeutic antitumor responses against B16 melanoma. These results suggest that CD4 T cells endowed with costimulatory ligands allow the design of effective vaccination strategies against cancer.

